# (NH_4_)_2_[UO_2_Cl_4_]·2H_2_O, a new uranyl tetra­chloride with ammonium charge-balancing cations

**DOI:** 10.1107/S2056989023005753

**Published:** 2023-07-07

**Authors:** Tsuyoshi A. Kohlgruber, Robert G. Surbella III

**Affiliations:** a Pacific Northwest National Laboratory, 902 Battelle Boulevard, Richland, WA, 99354, USA; University of Kentucky, USA

**Keywords:** ammonium, optical properties, uranyl tetra­chloride, X-ray diffraction, crystal structure

## Abstract

A new uranyl tetra­chloride salt with chemical formula, (NH_4_)_2_[UO_2_Cl_4_]·2H_2_O, namely, di­ammonium uranyl tetra­chloride dihydrate, **1**, was prepared and crystallized *via* slow evaporation from a solution of 2 *M* hydro­chloric acid. As confirmed by powder X-ray diffraction, the title compound crystallizes with an ammonium chloride impurity that formed as a result of the breakdown of a triazine precursor. The (UO_2_Cl_4_)^2−^ dianion is charge balanced by ammonium cations, while an extensive hydrogen-bond network donated from structural water mol­ecules stabilize the overall assembly. Compound **1** adds to the extensive collection of actinyl tetra­chloride salts, but it represents the first without an alkali cation for purely inorganic compounds. Diffuse reflectance and luminescence spectra show typical absorption and emission behavior, respectively, of uranyl materials.

## Abstract 2

While several actinyl tetra­halides have been synthesized and their structures reported, (NH_4_)_2_(UO_2_Cl_4_)·2H_2_O represents a new uranyl tetra­chloride salt synthesized in a slow evaporation from a 2 *M* hydro­chloric acid solution. Its optical properties were measured by diffuse reflectance and luminescence spectroscopies, while powder X-ray diffraction confirmed an ammonium chloride impurity phase.

## 1. Chemical context

Hexavalent actinides such as uranium, neptunium, and plutonium exist in aqueous solution as the linear triatomic actinyl cation, with formula (AnO_2_)^2+^. The actinyl ion coordinates a variety of poly- and mono-atomic anions such that four to six atoms bond in the equatorial plane (Burns, 2005 ▸; Lussier *et al.*, 2016 ▸). In part due to their ease of synthesis, structural simplicity, and high symmetry, the actinyl tetra­halide family of compounds has remained a relevant subclass of materials over several decades and has led to a deeper understanding of actinide electronic structure, bonding, and optical properties, among many others. The actinyl tetra­halides have general formula (*An*O_2_ *X*_4_)^2−^ (where *An* = U^VI^, Np^VI^, and Pu^VI^ and *X* = Cl^−^ and Br^−^) and have been studied to investigate periodic trends in *f*-element chemistry. Of the numerous compounds that include this anionic complex, the Cs^+^ salt with formula Cs_2_(AnO_2_Cl_4_) has been one of the most extensively characterized actinyl compounds. The uranyl structure was first reported in 1966 (Hall *et al.*, 1966 ▸) with an improved model reported in 1991 (Watkin *et al.*, 1991 ▸). In that time, it was used to qu­anti­tatively assign infrared (Ohwada, 1975 ▸) and Raman (Ohwada, 1980 ▸) active bands of the uranyl ion, which were found to be at 916 cm^−1^ and 831 cm^−1^, respectively. Improvements in analytical (*i.e.* X-ray absorption spectroscopies) and computational techniques (*i.e.* density functional theory calculations) over time have advanced our understanding in the electronic and mol­ecular orbital energies of the uranyl ion in Cs_2_(UO_2_Cl_4_), providing strong evidence that actinide atoms can bind with significant covalent character (Denning, 2007 ▸; Vitova *et al.*, 2015 ▸). Luminescence spectroscopy, Raman spectroscopy, and computational works have also been used to study bond-length changes of the uranyl ion with respect to different pressures in Cs_2_(UO_2_Cl_4_) (Osman *et al.*, 2016 ▸; Warzecha *et al.*, 2019 ▸). Beyond the Cs salt, systematic studies into actinyl bond strength changes as a function of metal center (*i.e. An* = U^VI^, Np^VI^ and Pu^VI^) have been reported for organic-based counter-cations (Schnaars & Wilson, 2013 ▸; Surbella III *et al.*, 2017 ▸; Schnaars & Wilson, 2018 ▸). Quite recently, focus has been placed on the cationic influence on supra­molecular assembly as well as actinyl bond-strength changes (Schnaars & Wilson, 2013 ▸; Surbella III *et al.*, 2016 ▸; Carter *et al.*, 2018 ▸; Pyrch *et al.*, 2020 ▸; Augustine *et al.*, 2023 ▸). Despite these numerous studies with actinyl tetra­halide species, we report a new inorganic uranyl tetra­chloride not charge-balanced by an alkali cation, with formula (NH_4_)_2_(UO_2_Cl_4_)·2H_2_O (compound **1**).

## 2. Structural commentary

**Figure 1:**
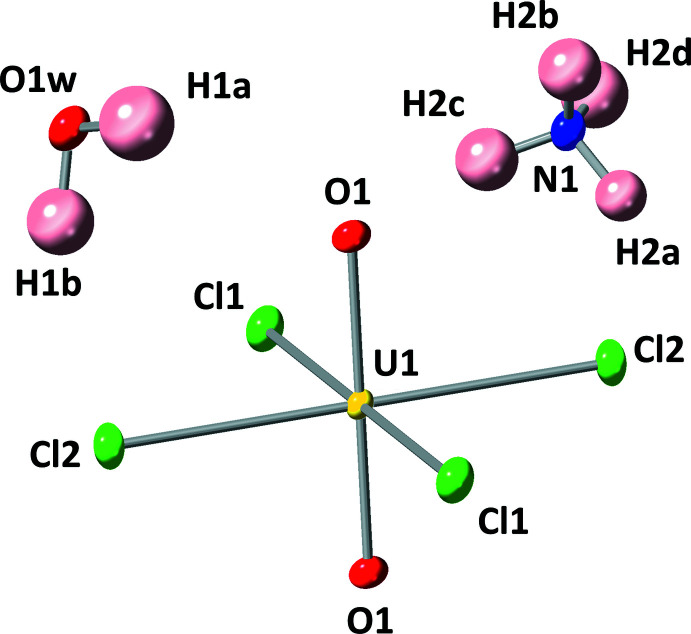
The uranyl tetra­chloride anionic unit along with a crystallographically unique water mol­ecule and ammonium cation. Displacement ellipsoids for non-hydrogen atoms are shown at 50% probability.

**Figure 2:**
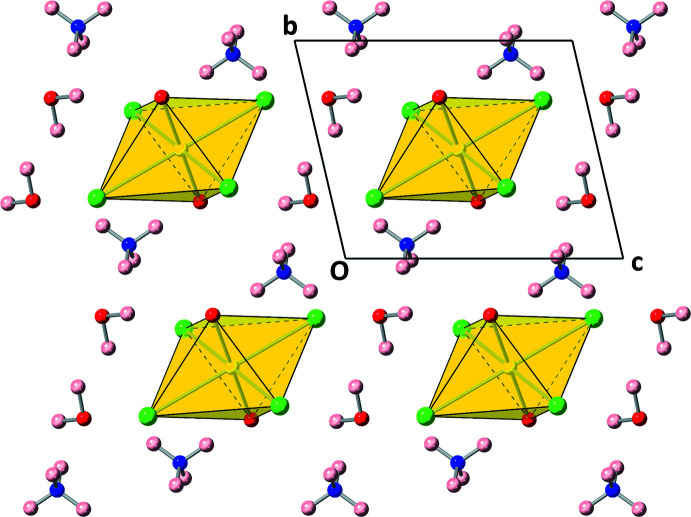
The crystal packing observed in compound **1** as viewed along the *a*-axis.

Compound **1** crystallizes in the space group *P* 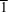 . The uranyl tetra­chloride dianion (UO_2_Cl_4_)^2−^ is composed of a U^VI^ metal center that is coordinated to two terminal, axial oxygen atoms and four equatorial chlorine atoms as shown in Fig. 1 ▸. The (UO_2_Cl_4_)^2−^ dianion adopts a square-bipyramidal coordination geometry with *D*_4*h*_ point group symmetry. The U^VI^ atom sits on a center of inversion symmetry, resulting in a linear uranyl (UO_2_)^2+^ cation with a U1—O1 bond distance of 1.7745 (14) Å and O1—U1—O1 angle of 180°. The U^VI^ atom is also coordinated to two crystallographically unique chlorine atoms with U1—Cl1 and U1—Cl2 bond distances of 2.6752 (5) Å and 2.6623 (4) Å, respectively. The two Cl1—U1—Cl2 bond angles measure 88.855 (15)° and 91.145 (15)°, and O1—U1—Cl1, bond angles also slightly deviate from 90°. The U—O (Lussier *et al.*, 2016 ▸) and U—Cl (Surbella III *et al.*, 2016 ▸) bond lengths are typical for these compounds. The structure contains one crystallographically unique structural water mol­ecule (O1*w*) with two O—H covalent bonds with restrained bond lengths near 0.95 Å, and one crystallographically unique ammonium cation (N1) is present to provide charge balance to the overall structure. There are four N—H covalent bonds with restrained bond lengths that are approximately 0.87 Å. The extended crystal structure is shown in Fig. 2 ▸.

## 3. Supra­molecular features

**Figure 3:**
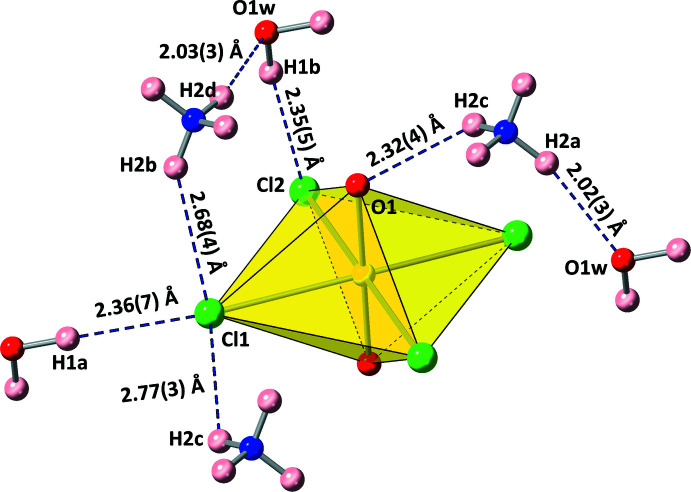
The seven unique hydrogen-bond inter­actions shown with the hydrogen-bond distances from donor hydrogen atom to acceptor atom.

**Figure 4:**
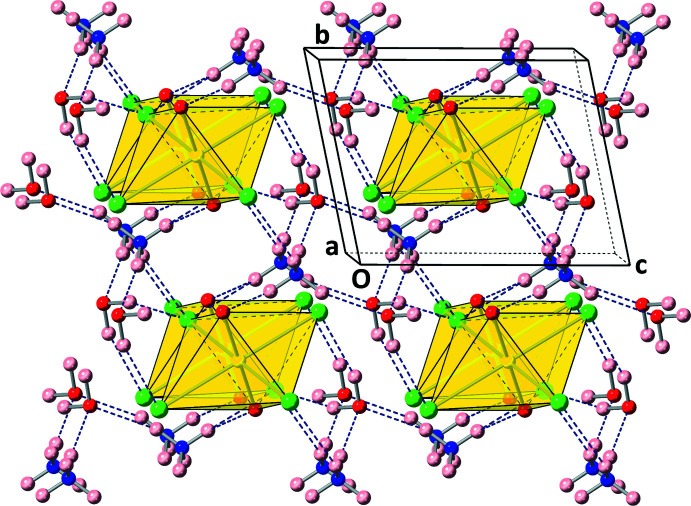
The crystal packing along with the hydrogen-bond network observed in compound **1** as viewed slightly offset along the *a*-axis.

A hydrogen-bond network consisting of seven unique inter­actions exists between ammonium cations, water mol­ecules, and uranyl tetra­chloride units as depicted in Fig. 3 ▸ and as tabulated in Table 1 ▸. Each water mol­ecule donates two hydrogen bonds *via* H1*A* and H1*B* donor atoms to two separate uranyl tetra­chloride complexes. On the other hand, each ammonium cation donates hydrogen bonds in three dimensions to three separate uranyl tetra­chloride units and two separate water mol­ecules, stabilizing the overall crystal structure into a complex network. Fig. 4 ▸ shows the hydrogen-bond network in the extended structure.

## 4. Database survey

Compound **1** is the first inorganic uranyl tetra­chloride charge-balanced with a non-alkali metal in the Inorganic Crystal Structure Database (Zagorac *et al.*, 2019 ▸). With respect to structures in the ICSD, Cs salts of the (AnO_2_Cl_4_)^2−^ species have been reported for U (Hall *et al.*, 1966 ▸; Watkin *et al.*, 1991 ▸; Tutov *et al.*, 1991 ▸; Schnaars & Wilson, 2013 ▸), Np (Wilkerson *et al.*, 2007 ▸), and Pu (Wilkerson & Scott, 2008 ▸; Schnaars & Wilson, 2013 ▸). Other charge-balancing cations reported in the ICSD for U^VI^ and Pu^VI^ include Rb (Anson *et al.*, 1996 ▸; Schnaars & Wilson, 2013 ▸) and tetra­methyl­ammonium (Schnaars & Wilson, 2013 ▸), while that of Np includes (UO_2_Cl_4_)^2−^-doped Np^VI^ (Wilkerson & Berg, 2009 ▸) and a mixed Np^V/VI^ oxidation state Cs salt (Alcock *et al.*, 1986 ▸). Although there is a tetra­methyl­ammonium salt in the ICSD, we consider it as a better member of the Crystal Structure Database (CSD) given the presence of organic-based (*i.e.* C—H bonds) components in the structure (Groom *et al.*, 2016 ▸).

With respect to the CSD, there are numerous reports with ammonium-based charge-balancing species (Di Sipio *et al.*, 1974 **a* ▸,b* ▸; Bois *et al.*, 1976 **a* ▸,b* ▸; Rogers *et al.*, 1987 ▸; Gatto *et al.*, 2004 ▸; Schnaars & Wilson, 2013 ▸; Biswas *et al.*, 2017 ▸; Serezkhina *et al.*, 2021 ▸). Compound **1** has ammonium with a water mol­ecule, while one report has ammonium with crown ethers (Rogers *et al.*, 1987 ▸). The other ammonium-based cations include organic-functional groups (Di Sipio *et al.*, 1974 **a* ▸,b* ▸; Bois *et al.*, 1976 **a* ▸,b* ▸; Gatto *et al.*, 2004 ▸; Schnaars & Wilson, 2013 ▸; Biswas *et al.*, 2017 ▸; Serezkhina *et al.*, 2021 ▸). Other types of cations that charge-balance (UO_2_Cl_4_)^2−^ in the CSD include pyridinium-based (Graziani *et al.*, 1975 ▸; Bombieri *et al.*, 1978 ▸; Marsh, 1988 ▸; Pospieszna *et al.*, 2008 ▸; Deifel & Cahill, 2009 ▸; Baker *et al.*, 2010 ▸; Andrews & Cahill, 2012 ▸; Lhoste *et al.*, 2013 ▸; Hashem *et al.*, 2013 ▸; Surbella III *et al.*, 2016 ▸, 2017 ▸; Carter *et al.*, 2018 ▸; Mishra *et al.*, 2019 ▸; Pyrch *et al.*, 2020 ▸), phenanthrolinium-based (Di Sipio *et al.*, 1981 ▸), imidazolium-based (Zalkin *et al.*, 1983 ▸; Qu *et al.*, 2014 ▸; Kohlgruber, 2022 ▸), and phospho­nium-based (Brown *et al.*, 1996 ▸; Schnaars & Wilson, 2014 ▸) species. Other (UO_2_Cl_4_)^2−^ complexes have crystallized in the presence of separate metal complexes (Moody & Ryan, 1979 ▸; Rogers *et al.*, 1987 ▸, 1990 ▸; Pons y Moll *et al.*, 2001 ▸; Hashem *et al.*, 2014 ▸; Falaise *et al.*, 2015 ▸; Zhang *et al.*, 2017 ▸; Schöne *et al.*, 2018 ▸), crown ethers (Wang *et al.*, 1986 ▸; Rogers *et al.*, 1987 ▸, 1991 ▸; Rogers & Benning, 1991 ▸; Evans *et al.*, 2002 ▸) and porphyrins (Mishra *et al.*, 2019 ▸). In total, there are over 60 known uranyl tetra­chloride crystal structures in the CSD. Reference codes for these compounds can be found in the supporting information.

## 5. Synthesis and crystallization

Concentrated hydro­chloric acid, HCl, (Sigma-Aldrich, 37%) was diluted to 2 *M*. Then, 0.0366 g (0.44 mmol) of 1,3,5-triazine (Sigma-Aldrich, 97.0%) was dissolved into 1 mL of 2 *M* HCl in a 1-dram borosilicate glass reaction vial. Uranyl acetate dihydrate (0.10216 g; 2.4 mmol) was added to this solution and allowed to dissolve completely. The vial was placed uncapped in a 20 mL centrifuge tube on a bed of desiccant. The centrifuge tube was capped, and the reaction solution was allowed to evaporate for 3 weeks until large yellow crystals formed. It was noticed that compound **1** partially dissolves in ethanol, affecting the preparation for characterization beyond single-crystal X-ray diffraction. Powder-diffraction data was collected using a Rigaku Ultima IV Diffractometer with Cu *Kα* radiation and a linear position-sensitive detector. The analysis revealed an ammonium chloride, NH_4_Cl, impurity phase along with compound **1**. Diffuse reflectance and luminescence spectra were also collected for the mixed-phase material and can be found in the supporting information along with the powder-diffraction data.

## 6. Refinement

Crystal data, data collection, and structure refinement details are summarized in Table 2 ▸. All non-hydrogen atoms were refined anisotropically. All hydrogen atoms were found in Fourier difference maps, and their positions refined with positional restraints.

## Supplementary Material

Crystal structure: contains datablock(s) I. DOI: 10.1107/S2056989023005753/pk2687sup1.cif

Structure factors: contains datablock(s) I. DOI: 10.1107/S2056989023005753/pk2687Isup2.hkl

Thermal Ellipsoidal plots and data, (e.g., Powder X-ray diffraction, diffuse reflectance spectroscopy, and photo luminescence spectroscopy). DOI: 10.1107/S2056989023005753/pk2687sup3.docx

CCDC reference: 2278035

Additional supporting information:  crystallographic information; 3D view; checkCIF report

## Figures and Tables

**Table 1:** Hydrogen-bond geometry (Å, °)

*D*—H⋯*A*	*D*—H	H⋯*A*	*D*⋯*A*	*D*—H⋯*A*
O1w—H1*A*⋯Cl1^i^	0.95 (6)	2.36 (7)	3.283 (2)	165 (7)
O1w—H1*B*⋯Cl2^ii^	0.95 (5)	2.35 (5)	3.268 (2)	163 (5)
N1—H2*A*⋯O1w	0.87 (3)	2.02 (3)	2.843 (2)	157 (3)
N1—H2*B*⋯Cl1^iii^	0.87 (4)	2.68 (4)	3.441 (2)	148 (3)
N1—H2*C*⋯O1	0.87 (2)	2.32 (4)	3.014 (3)	137 (4)
N1—H2*C*⋯Cl1^iv^	0.87 (2)	2.77 (3)	3.4060 (17)	131 (4)
N1—H2*D*⋯O1w^v^	0.87 (3)	2.03 (3)	2.887 (3)	169 (3)

Symmetry codes: (i) 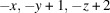 ; (ii) 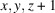 ; (iii) 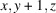 ; (iv) 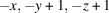 ; (v) 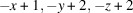 .

**Table 2:** Experimental details

Crystal data	
Chemical formula	(NH_4_)_2_[UO_2_Cl_4_]·2H_2_O
*M*_r_	483.95
Crystal system, space group	Triclinic, *P* 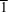
Temperature (K)	100
*a*, *b*, *c* (Å)	6.6574 (4), 6.6954 (4), 7.4018 (4)
α, β, γ (°)	99.827 (2), 93.879 (2), 117.354 (1)
*V* (Å^3^)	284.69 (3)
*Z*	1
Radiation type	Mo *K*α
μ (mm^−1^)	15.17
Crystal size (mm)	0.10 × 0.03 × 0.03

Data collection	
Diffractometer	Bruker D8 Venture
Absorption correction	Multi-scan (*SADABS*; Krause *et al.*, 2015 ▸)
*T*_min_, *T*_max_	0.460, 0.747
No. of measured, independent and observed [*I* > 2σ(*I*)] reflections	24049, 2803, 2803
*R*_int_	0.042
(sin θ/λ)_max_ (Å^−1^)	0.842

Refinement	
*R*[*F* ^2^ > 2σ(*F* ^2^)], *wR*(*F* ^2^), *S*	0.017, 0.041, 1.09
No. of reflections	2803
No. of parameters	76
No. of restraints	12
H-atom treatment	All H-atom parameters refined
Δρ_max_, Δρ_min_ (e Å^−3^)	2.94, −1.97

Computer programs: *APEX4* and *SAINT* (Bruker, 2014 ▸), *SHELXT* (Sheldrick, 2015*a* ▸), *SHELXL* (Sheldrick, 2015*b* ▸), *CrystalMaker* (*CrystalMaker*, 2014 ▸), and *publCIF* (Westrip, 2010 ▸).

## supplementary crystallographic information

### Crystal data

**Table:**

(NH_4_)_2_[UO_2_Cl_4_]·2H_2_O	*Z* = 1
*M**_r_* = 483.95	*F*(000) = 218
Triclinic, *P*1	*D*_x_ = 2.823 Mg m^−^^3^
*a* = 6.6574 (4) Å	Mo *K*α radiation, λ = 0.71073 Å
*b* = 6.6954 (4) Å	Cell parameters from 9764 reflections
*c* = 7.4018 (4) Å	θ = 3.5–36.7°
α = 99.827 (2)°	µ = 15.17 mm^−^^1^
β = 93.879 (2)°	*T* = 100 K
γ = 117.354 (1)°	Block, yellow
*V* = 284.69 (3) Å^3^	0.10 × 0.03 × 0.03 mm

### Data collection

**Table:**

Bruker D8 Venture diffractometer	2803 reflections with *I* > 2σ(*I*)
Radiation source: microsource Diamond II	*R*_int_ = 0.042
φ and ω scans	θ_max_ = 36.8°, θ_min_ = 3.5°
Absorption correction: multi-scan (SADABS; Krause *et al.*, 2015)	*h* = −11→11
*T*_min_ = 0.460, *T*_max_ = 0.747	*k* = −11→11
24049 measured reflections	*l* = −12→12
2803 independent reflections	

### Refinement

**Table:**

Refinement on *F*^2^	Primary atom site location: dual
Least-squares matrix: full	Secondary atom site location: difference Fourier map
*R*[*F*^2^ > 2σ(*F*^2^)] = 0.017	Hydrogen site location: difference Fourier map
*wR*(*F*^2^) = 0.041	All H-atom parameters refined
*S* = 1.09	*w* = 1/[σ^2^(*F*_o_^2^) + (0.0306*P*)^2^] where *P* = (*F*_o_^2^ + 2*F*_c_^2^)/3
2803 reflections	(Δ/σ)_max_ = 0.001
76 parameters	Δρ_max_ = 2.94 e Å^−^^3^
12 restraints	Δρ_min_ = −1.97 e Å^−^^3^

### Special details

**Table:**

Geometry. All estimated standard deviations (esds), except those pertaining to the dihedral angle between two least squares (ls) planes, are estimated using the full covariance matrix. All esds (except the esd in the dihedral angle between two l.s. planes) are estimated using the full covariance matrix. The cell esds are taken into account individually in the estimation of esds in distances, angles and torsion angles; correlations between esds in cell parameters are only used when they are defined by crystal symmetry. An approximate (isotropic) treatment of cell esds is used for estimating esds involving l.s. planes.

### Fractional atomic coordinates and isotropic or equivalent isotropic displacement parameters (Å^2^)

**Table:**

	*x*	*y*	*z*	*U*_iso_*/*U*_eq_	
U1	0.500000	0.500000	0.500000	0.00924 (3)	
Cl1	0.11039 (7)	0.32334 (9)	0.63968 (6)	0.01683 (7)	
Cl2	0.27372 (8)	0.27569 (9)	0.15652 (6)	0.01779 (7)	
O1	0.4614 (3)	0.7395 (3)	0.4746 (2)	0.0157 (2)	
O1W	0.2602 (3)	0.7346 (3)	1.0758 (2)	0.0193 (2)	
H1A	0.173 (10)	0.747 (13)	1.169 (7)	0.060 (19)*	
H1B	0.253 (11)	0.589 (5)	1.071 (9)	0.048 (16)*	
N1	0.2678 (3)	0.9378 (3)	0.7673 (2)	0.0165 (2)	
H2A	0.227 (6)	0.850 (5)	0.846 (4)	0.028 (11)*	
H2B	0.186 (5)	1.008 (6)	0.765 (5)	0.042 (14)*	
H2C	0.247 (7)	0.855 (6)	0.657 (2)	0.042 (14)*	
H2D	0.412 (2)	1.040 (5)	0.800 (5)	0.049 (16)*	

### Atomic displacement parameters (Å^2^)

**Table:**

	*U*^11^	*U*^22^	*U*^33^	*U*^12^	*U*^13^	*U*^23^
U1	0.01069 (4)	0.01103 (4)	0.00762 (4)	0.00584 (3)	0.00291 (2)	0.00367 (2)
Cl1	0.01357 (14)	0.02264 (19)	0.01467 (15)	0.00771 (14)	0.00622 (12)	0.00655 (14)
Cl2	0.01902 (16)	0.02084 (18)	0.01029 (14)	0.00814 (14)	0.00083 (12)	0.00047 (13)
O1	0.0210 (5)	0.0150 (5)	0.0159 (5)	0.0113 (5)	0.0057 (4)	0.0067 (4)
O1W	0.0189 (6)	0.0234 (7)	0.0175 (6)	0.0096 (5)	0.0063 (5)	0.0093 (5)
N1	0.0180 (6)	0.0189 (6)	0.0126 (5)	0.0085 (5)	0.0027 (5)	0.0045 (5)

### Geometric parameters (Å, º)

**Table:**

U1—O1	1.7745 (14)	O1W—H1A	0.948 (10)
U1—O1^i^	1.7745 (14)	O1W—H1B	0.947 (10)
U1—Cl2	2.6623 (4)	N1—H2A	0.868 (9)
U1—Cl2^i^	2.6623 (4)	N1—H2B	0.872 (9)
U1—Cl1	2.6752 (5)	N1—H2C	0.870 (9)
U1—Cl1^i^	2.6752 (5)	N1—H2D	0.871 (9)
			
O1—U1—O1^i^	180.0	O1^i^—U1—Cl1^i^	89.87 (5)
O1—U1—Cl2	90.32 (5)	Cl2—U1—Cl1^i^	88.855 (15)
O1^i^—U1—Cl2	89.68 (5)	Cl2^i^—U1—Cl1^i^	91.145 (15)
O1—U1—Cl2^i^	89.68 (5)	Cl1—U1—Cl1^i^	180.000 (17)
O1^i^—U1—Cl2^i^	90.32 (5)	H1A—O1W—H1B	104 (6)
Cl2—U1—Cl2^i^	180.000 (11)	H2A—N1—H2B	109.7 (14)
O1—U1—Cl1	89.86 (5)	H2A—N1—H2C	109.8 (14)
O1^i^—U1—Cl1	90.13 (5)	H2B—N1—H2C	109.1 (14)
Cl2—U1—Cl1	91.145 (15)	H2A—N1—H2D	109.7 (14)
Cl2^i^—U1—Cl1	88.855 (15)	H2B—N1—H2D	109.1 (14)
O1—U1—Cl1^i^	90.14 (5)	H2C—N1—H2D	109.5 (14)

Symmetry code: (i) −*x*+1, −*y*+1, −*z*+1.

### Hydrogen-bond geometry (Å, º)

**Table:**

*D*—H···*A*	*D*—H	H···*A*	*D*···*A*	*D*—H···*A*
O1w—H1*A*···Cl1^ii^	0.95 (6)	2.36 (7)	3.283 (2)	165 (7)
O1w—H1*B*···Cl2^iii^	0.95 (5)	2.35 (5)	3.268 (2)	163 (5)
N1—H2*A*···O1w	0.87 (3)	2.02 (3)	2.843 (2)	157 (3)
N1—H2*B*···Cl1^iv^	0.87 (4)	2.68 (4)	3.441 (2)	148 (3)
N1—H2*C*···O1	0.87 (2)	2.32 (4)	3.014 (3)	137 (4)
N1—H2*C*···Cl1^v^	0.87 (2)	2.77 (3)	3.4060 (17)	131 (4)
N1—H2*D*···O1w^vi^	0.87 (3)	2.03 (3)	2.887 (3)	169 (3)

Symmetry codes: (ii) −*x*, −*y*+1, −*z*+2; (iii) *x*, *y*, *z*+1; (iv) *x*, *y*+1, *z*; (v) −*x*, −*y*+1, −*z*+1; (vi) −*x*+1, −*y*+2, −*z*+2.
