# Supplementary material for: (NH4)2[UO2Cl4]·2H2O, a new uranyl tetra­chloride with ammonium charge-balancing cations
Source: Acta Crystallogr E Crystallogr Commun. 2023 Jul 7;79(Pt 8):702–6. doi: 10.1107/S2056989023005753 (PMC10439412; doi:10.1107/S2056989023005753)
Supplement: Supplementary file 3 [file e-79-00702-sup3.docx]

**Supplementary Information**

**for**

**(NH_4_)_2_(UO_2_Cl_4_)·2H_2_O, a new uranyl tetrachloride with ammonium charge-balancing cations**

Tsuyoshi A. Kohlgruber,^a^ and Robert G. Surbella III^a,^*

^a^ Pacific Northwest National Laboratory, 902 Battelle Boulevard, Richland, WA 99354, USA

* Email: robert.surbella@pnnl.gov

**Supporting Information**

**Table of Contents**:

1. Thermal ellipsoid model
2. Powder X-ray Diffraction
3. Diffuse reflectance spectroscopy
4. Luminescence measurements
5. References
6. Thermal ellipsoid model


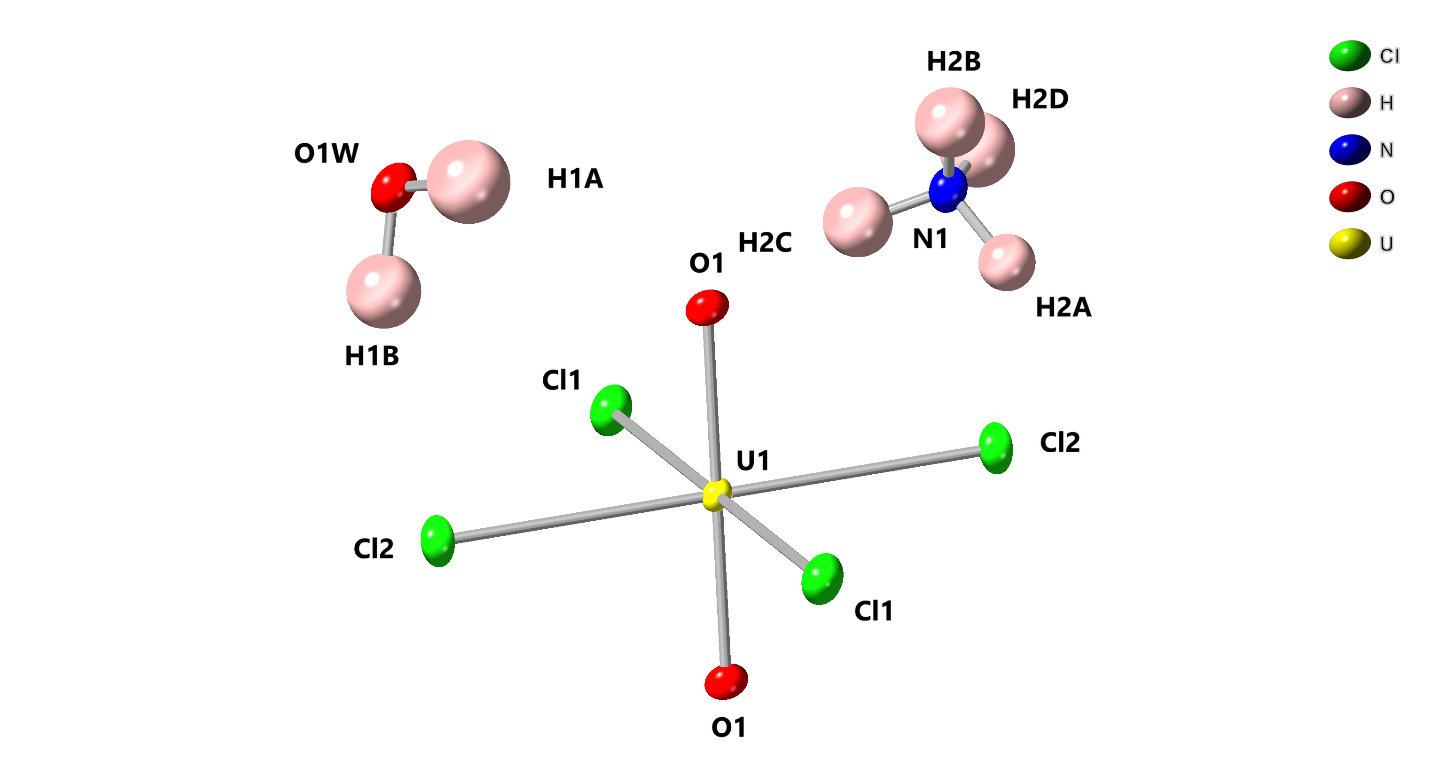


**Figure S1**. Thermal ellipsoid model shown at 50% probability of compound **1**.

1. Powder X-ray diffraction (pXRD)

Powder X-ray diffraction was collected on bulk sample of compound **1** to determine if the material is phase pure. Diffraction data was collected using a Rigaku Ultima IV Diffractometer with Cu Kα radiation and a linear position sensitive detector. The pattern was obtained with instrumental setup of 5 mm divergence slit and a Ni foil filter to reduce Kβ contributions. The scan was performed from 10-60° 2θ with a step size of 0.02m and scan rate of 2° per min. The experimental diffractogram was compared to a calculated pattern from the crystal structure obtained from the single-crystal X-ray diffraction experiment. The Diffrac.EVA software was used to help identify unknown peaks, matching them to ammonium chloride, NH_4_Cl (Bruker, 2018). The deposited crystallographic information file (CIF) for NH_4_Cl with collection code 20682 was obtained from the Inorganic Crystal Structure Database (ICSD) and was used to calculate a pattern. Figure S2 shows the experimental powder pattern compared to the calculated patterns for compound **1** and NH_4_Cl, which confirms the bulk material is a mixture of the two phases.


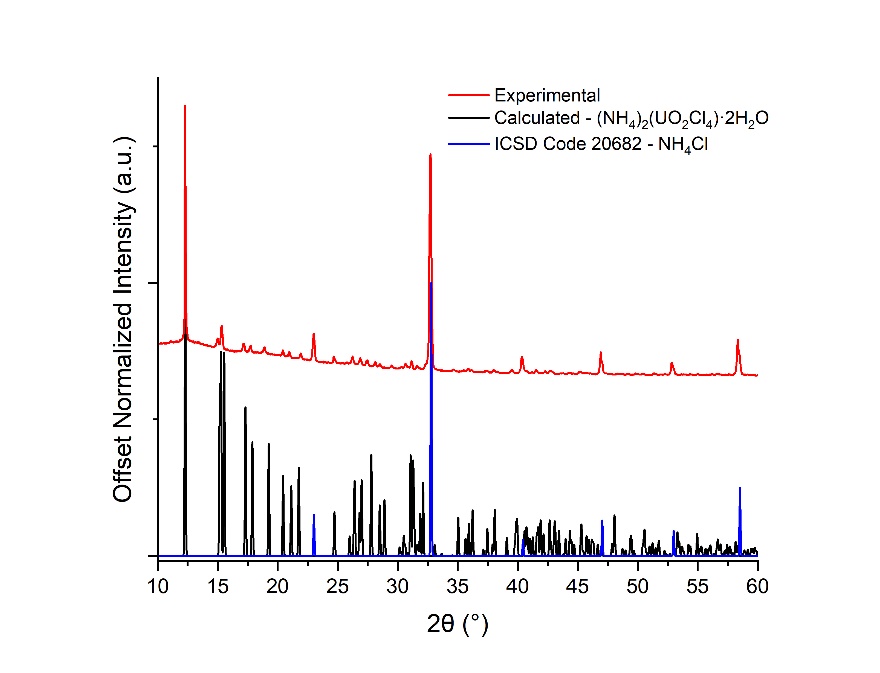


**Figure S2**. The red trace is the experimental pXRD pattern of the bulk material. The black and blue traces are the calculated powder patterns from the CIFs for compound **1**, (NH_4_)_2_(UO_2_Cl_4_)·2H_2_O, and NH_4_Cl. The NH_4_Cl CIF was obtained from the ICSD with collection code 20682.

1. Diffuse Reflectance Spectroscopy (DRS)

Bulk material of compound **1** was loaded into an Agilent UV-vis-NIR powder cell holder containing an optical quartz window and backfilled with barium sulfate (BaSO_4_). A diffuse reflectance spectrum was collected at 298K for bulk material using a Cary-5000 equipped with a DRA-2500 external diffuse reflectance accessory. Three scans were measured from 200-800 nm at a scan rate of 100 nm per min in reflectance mode. The scans were background subtracted for BaSO_4_. The average of the 3 scans is presented in Figure S3. A charge transfer band is observed centered around approximately 450 nm, which is typical of uranyl materials (O_yl_ 🡪 U) (Nockemann *et al.*, 2007). There is a second equatorial (Cl 🡪 U) charge transfer band centered around 300 nm (Denning et al., 1976).


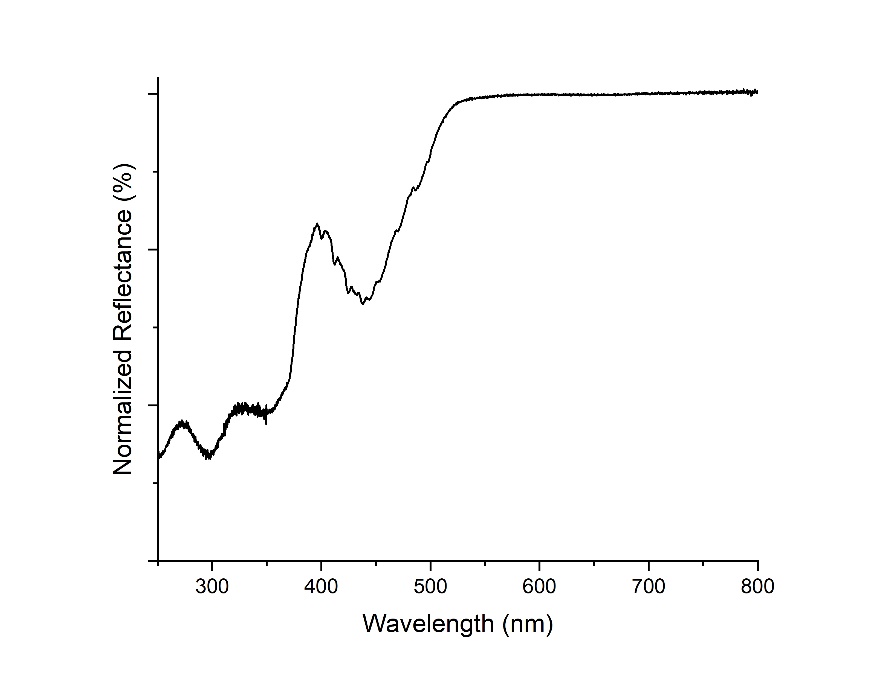


**Figure S3**. The room temperature diffuse reflectance spectrum for the mixed phase material containing compound **1**, (NH_4_)_2_(UO_2_Cl_4_)·2H_2_O, and NH_4_Cl.

1. Luminescence Measurements

Samples were wet mounted with ethanol onto a quartz plate and material sealed with a black mount backing. Due to the soluble nature of compound **1**, the luminescence sample was mostly white powder inferred to be majority NH_4_Cl. Luminescence measurements were collected using a Horiba Nano-log equipped with a 450 W Xe arc lamp excitation source and UV-Visible photomultiplier tube emission detector (185-850 nm). Instrument parameters were optimized for both the excitation and emission prior to obtaining the scans as presented in Figure S4. The FluorEssence software package was used to obtain and analyze data. The emission profile is typical of uranyl tetrachlorides and exhibits a vibronically coupled green emission with resolved peaks around 502, 524, and 548 nm (Nockemann *et al.*, 2007; Surbella III *et al.*, 2016). The excitation profile bears resemblance to that of the absorption bands observed by DRS, with two charge transfer bands centered around 321 and 423 nm. The peak at 263 nm is the lamp harmonic.


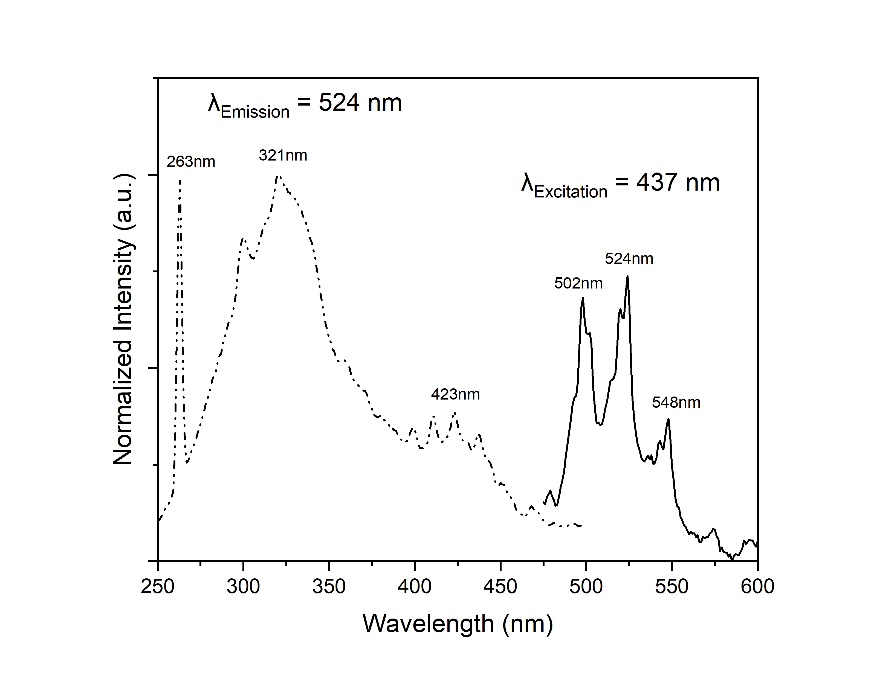


**Figure S4**. The room temperature luminescence emission and excitation spectra for the mixed phase material containing compound **1**, (NH_4_)_2_(UO_2_Cl_4_)·2H_2_O, and NH_4_Cl. The emission spectrum was collected with a 437 nm excitation, while the excitation spectrum was collected with a 524 nm emission wavelength. The peak at 263 nm is an artifact of Rayleigh scattering.

1. References

Bruker (2018). *DIFFRAC.EVA: software to evaluate X-ray diffraction data.* Version 6.0. http://www.bruker.eva

Denning, R. G., Snellgrove, T. R., Woodwark, D. R. (1976). *Mol. Phys.* pp. 419-442.

Nockemann, P., Servaes, K., Deun, R. V., Hecke, K. V., Meervelt, L. V., Binnemans, K., Görller-Walrand, C. (2007). *Inorg. Chem.* pp. 11335-11344.

Surbella III, R. G., Andrews, M. B., Cahill, C. L. (2016). *J. Solid State Chem.* pp. 235-271.
